# Drug transport in brain *via *the cerebrospinal fluid

**DOI:** 10.1186/2045-8118-8-7

**Published:** 2011-01-18

**Authors:** William M Pardridge

**Affiliations:** 1 Department of Medicine, UCLA, Los Angeles, CA 90024, USA

## Abstract

The human brain has no lymphatic system, but produces over a half-liter each day of cerebrospinal fluid. The cerebrospinal fluid is secreted at the choroid plexus and occupies the cavities of the four ventricles, as well as the cranial and spinal sub-arachnoid space. The cerebrospinal fluid moves over the surfaces of the brain and spinal cord and is rapidly absorbed into the general circulation. The choroid plexus forms the blood-cerebrospinal fluid barrier, and this barrier is functionally distinct from the brain microvascular endothelium, which forms the blood-brain barrier. Virtually all non-cellular substances in blood distribute into cerebrospinal fluid, and drug entry into cerebrospinal fluid is not an index of drug transport across the blood-brain barrier. Drug injected into the cerebrospinal fluid rapidly moves into the blood via bulk flow, but penetrates into brain tissue poorly owing to the limitations of diffusion. Drug transport into cerebrospinal fluid vs. brain interstitial fluid requires knowledge of the relative expression of transporters at the choroid plexus *versus *the brain microvascular endothelium.

## Introduction

One hundred years ago, the prevailing view was that nutrients in blood were transferred to brain not *via *the vascular system, but rather *via *cerebrospinal fluid (CSF). This idea emerged from studies with vital dyes, which were acidic such as trypan blue, and the acidic dyes did not penetrate the vasculature, which forms the blood-brain barrier (BBB). It was not until the 1930s, when studies with basic vital dyes, which do cross the vasculature, demonstrated the importance of the vascular route to brain. Nevertheless, the misconceptions of a hundred years ago are alive and well today. Virtually all neurologists, academic neuroscientists, and industry scientists believe that drug entry into CSF is a measure of drug penetration through the BBB. It is also widely believed that the trans-cranial delivery of drug to the CSF compartment is a viable solution to the brain drug delivery problem.

There are two barrier systems in brain: the BBB, which is formed by the brain microvascular endothelium, and the blood-CSF barrier, which is formed by the choroid plexus epithelium. In addition, the arachnoid epithelium forms a barrier around the brain creating the sub-arachnoid space. Drug transport from blood to CSF is regulated by the choroid plexus, or blood-CSF barrier. Drug transport from blood to interstitial fluid (ISF) is regulated by the brain microvascular endothelium, or BBB. In order to delineate the relative roles of the choroid plexus and the BBB in limiting drug transport in the brain, it is useful to consider the quantitative aspects of CSF flow within the 1200 g human brain.

### Anatomical considerations

There is approximately 140 mL of CSF in the human brain [1], which fills the four ventricles (20 mL), spinal sub-arachnoid space (30 mL), and the cranial sub-arachnoid space (90 mL). The CSF volume in rat brain is approximately 90 μL [2]. In the human brain, the entire CSF volume is produced and excreted to blood every 4-5 hours or 4-5 times per day. CSF is secreted by the choroid plexus epithelium and passes from the lateral ventricles to the third ventricle to the fourth ventricle to the cranial and spinal subarachnoid spaces, and finally the CSF is absorbed into superior sagittal sinus of the peripheral bloodstream across the arachnoid villi. The estimated surface area of the human choroid plexus is approximately 0.021 m^2 ^[3], which is many-fold less than the surface area of the brain capillary endothelium which forms the BBB. With respect to the vasculature of brain, there are > 100 billion capillaries in the human brain comprising a total length of approximately 400 miles. Yet, the intra-endothelial volume is quite small, about 1 uL/g brain. Thus, the volume of the capillary intracellular space in brain is about 1 uL for the rat brain, and about 1 mL for the human brain. The microvasculature in the human brain is dense. The inter-capillary distance in brain is about 40 μm, which is space for 2 neurons. Thus, every neuron in the brain is perfused by its own blood vessel. Diffusion across a distance of 20 μm is instantaneous, even for a large molecule. Once the drug or solute crosses the vascular barrier from blood there is immediate equilibration throughout the entire brain extravascular volume.

### Drug entry into CSF is not a measure of BBB permeability

Drug entry into the CSF compartment from blood should not be taken as an index of drug transport across the BBB. Drug transfer from blood to CSF occurs across the choroid plexus, which is leaky compared to the BBB. Virtually all small and large molecules in blood penetrate into CSF, at a rate inversely related to the molecular weight of the substance. The finding of drug entry into CSF is expected, and does not discriminate between those drugs that do, and do not, cross the BBB. This is because the choroid plexus, which regulates drug transfer into CSF from blood, and the brain capillary endothelium, which regulates drug transfer into ISF from blood, are comprised of completely different epithelial/endothelial barriers. The application of recent proteomics technology will enable the description of the different transporter expression profiles in the BBB and choroid plexus [4]. The difference between drug transfer across the choroid plexus and across the BBB is illustrated in the case of azidothymidine (AZT). This drug is rapidly transported across the choroid plexus epithelium and rapidly enters the CSF compartment in humans [5]. However, there is negligible transport of AZT across the BBB *in vivo*, owing to active efflux transport processes at the brain microvasculature [6].

### Drug injection into the CSF is a slow intravenous infusion

Drug that is injected into the CSF compartment is rapidly transported out of brain to the blood. Following the ICV injection of drug, it moves through the CSF flow tracks, and is absorbed into the peripheral bloodstream across the arachnoid villi to enter the general circulation. This is illustrated by barbiturate studies in the dog. The dose of barbiturate that causes anesthesia in the dog is the same, whether the drug is given by an intravenous or intra-cerebroventricular (ICV) route [7]. The barbiturate injected into the CSF exits the brain, rapidly enters the peripheral blood, and then re-enters the brain across the BBB to induce anesthesia, similar to the pathway taken by drug injected intravenously. The ICV administration of cholecystokinin (CCK) decreases feeding behavior, not via a central mechanism within the brain, but rather due to entry of the neuropeptide into the peripheral bloodstream and activation of CCK receptors in peripheral tissues [8]. Christy and Fishman [9] noted 50 years ago in studies of CSF transport of cortisol in the dog that, "a CSF injection is like a slow intravenous infusion."

### Drug penetration into brain parenchyma from CSF is minimal

When drug is injected into the CSF compartment, the drug exits this space relatively rapidly via convection and bulk flow through the CSF flow tracts. Conversely, the entry of drug into brain parenchyma from the CSF compartment is mediated by diffusion, a process that is much slower than CSF convection and bulk flow. Moreover, diffusion decreases exponentially with the distance. It takes four days for albumin to diffuse 5 mm through water [10]. During four days, the entire CSF volume has turned over about 20 times. The effective diffusion distance in the brain is decreased with binding of the drug to brain cell surfaces, efflux across the BBB, and metabolism of the drug. If the concentration of drug is measured at each mm of brain parenchyma removed from the ependymal surface surrounding the ventricular CSF, one finds that the drug concentration in brain parenchyma decreases logarithmically as the distance from the CSF surface is increased [11]. The maximum penetration of a relatively inert small molecule such as hydroxyurea into the brain is 2 mm, whereas the maximal penetration of a biologically active neuropeptide such as brain-derived neurotrophic factor (BDNF) is only 0.3 mm [12]. The concentration of a small molecule such as 1,3-bis(2-chloroethyl-1-nitroso-urea) (BCNU) in brain parenchyma removed just 1 mm from the CSF surface is only 1% of the BCNU concentration in the CSF compartment [13]. In practice, most investigators do not measure drug concentration in brain at 1 or 2 mm from the CSF compartment, but rather measure drug concentration in the entire brain parenchyma ipsilateral to the CSF injection. When this is done, it is difficult to measure any increase in the concentration of the drug in brain of rats or monkeys [14,15], because the drug rapidly exits the CSF to the general circulation following an ICV injection.

### CSF administration exposes the ependymal surface to high drug concentrations

Since the penetration of drug into brain decreases exponentially with the distance from the CSF surface [11], it is necessary to administer high concentrations of drug into the CSF compartment. The ependymal surface is exposed to very high drug concentrations, which can have toxic side effects. The ICV administration of nerve growth factor (NGF) resulted in axonal sprouting and Schwann cell hyperplasia on the ependymal or arachnoid surface [16]. The ICV administration of fibroblast growth factor (FGF-2) results in peri-ventricular astrogliosis [17]. The ICV administration of glial-derived neurotrophic factor (GDNF) in Parkinson's disease, resulted in no penetration of the neuropeptide into the substantia nigra or into the caudate putamen nucleus, but did result in a high incidence of adverse events [18]. Conversely, it is sometimes desirable to deliver high concentrations of drug to the meningeal surface of the brain, such as in the treatment of meningeal infiltration of leukemia cells, and this can be achieved with intrathecal drug administration.

### The CSF "microcirculation"

The CSF 'macrocirculation' involves production of the CSF within the ventricles, bulk flow through the subarachnoid space, and absorption into the arachnoid villi. However, along this route there is a CSF 'microcirculation' that originates from the pial surface of the brain [19]. There is evidence for solute movement from CSF to brain parenchyma via transport through the Virchow-Robin spaces surrounding the penetrating arteries at the pial surface. From there, CSF components may move along the basement membrane of parenchymal capillaries via a process of bulk flow driven by the pulsations of penetrating arteries in the brain. The CSF microcirculation provides a pathway for solute movement from CSF to deep spaces within brain parenchyma. However, the CSF microcirculation is not a quantitatively important pathway for drug distribution into the brain from the CSF compartment. This is because the rate of bulk flow of fluid through the Virchow-Robin spaces from the pial surface is slow compared to the rapid rate of bulk flow through the CSF macrocirculation. The rate of bulk flow of CSF via the macrocirculation is 520 and 2 μL/min per whole brain in humans and rats, respectively [2]. The rate of CSF bulk flow via the macrocirculation is approximately 20-fold greater than the rate of bulk flow through the parenchyma via the microcirculation, which is approximately 0.1 μL/min in the rat [20].

The CSF macrocirculation functions as a closed hydraulic system, which provides a mechanical buffer between the skull and the brain, and relieves the effects of gravitational and accelerational stresses [21]. Without the CSF system, the brain could not function and brain abnormalities follow from either the under- or over-production of CSF. Since the entire CSF volume is turned over 4-5 times each day, it is crucial that the choroid plexus epithelial transport processes that underlie the production of CSF be tightly regulated. A greater understanding of this regulation will arise from the elucidation of the molecular and cellular biology of solute transporters at both the apical and basolateral membranes of the choroid plexus epithelial cell.

## Conclusions

Drug entry into CSF from blood is expected for all drugs, irrespective of whether the drug crosses the BBB. The finding of drug penetration into the CSF should not be taken as evidence that the drug crosses the BBB. Drug injected into the CSF compartment is rapidly exported to the blood with minimal penetration of brain parenchyma more than 1-2 mm from the ependymal surface of brain.

## Competing interests

The author is consultant to ArmaGen Technologies, Inc.

## Authors' contributions

Sole author and had read and approved the final version of the manuscript.

## References

[B1] Oldendorf WH Cerebrospinal fluid formation and circulation Prog Nuc Med 1972 1 336 358 4630864

[B2] Davson H The Cerebrospinal Fluid Handbook of Neurochemistry 1969 2 23 48

[B3] Dohrmann GJ The choroid plexus: a historical review Brain Res 1970 18 197 218 10.1016/0006-8993(70)90324-0 4929003

[B4] Kamiie J Ohtsuki S Iwase R Ohmine K Katsukura Y Yanai K Sekine Y Uchida Y Ito S Terasaki T Quantitative atlas of membrane transporter proteins: development and application of a highly sensitive simultaneous LC/MS/MS method combined with novel *in-silico *peptide selection criteria Pharm Res 2008 25 1469 1483 10.1007/s11095-008-9532-4 18219561

[B5] Yarchoan R Broder S Development of antiretroviral therapy for the acquired immunodeficiency syndrome and related disorders N Engl J Med 1987 316 557 564 10.1056/NEJM198702263160925 3543683

[B6] Dykstra KH Arya A Arriola DM Bungay PM Morrison PF Dedrick RL Microdialysis study of zidovudine (AZT) transport in rat brain J Pharmacol Exp Ther 1993 267 1227 1236 8263784

[B7] Aird RB A study of intrathecal, cerebrospinal fluid-to-brain exchange Exp Neurol 1984 86 342 358 10.1016/0014-4886(84)90192-4 6548450

[B8] Crawley JN Fiske SM Durieux C Derrien M Roques BP Centrally administered cholecystokinin suppresses feeding through a peripheral-type receptor mechanism J Pharmacol Exp Ther 1991 257 1076 1080 2046021

[B9] Christy NP Fishman RA Studies of the blood-cerebrospinal fluid barrier to cortisol in the dog J Clin Invest 1961 40 1997 2006 10.1172/JCI104426 13879330 PMC290904

[B10] Pardridge WM Peptide Drug Delivery to the Brain 1991 New York: Raven Press

[B11] Blasberg RG Patlak C Fenstermacher JD Intrathecal chemotherapy: brain tissue profiles after ventriculocisternal perfusion J Pharmacol Exp Ther 1975 195 73 83 810575

[B12] Mak M Fung L Strasser JF Saltzman WM Distribution of drugs following controlled delivery to the brain interstitium J Neurooncol 1995 26 91 102 10.1007/BF01060215 8787851

[B13] Fung LK Shin M Tyler B Brem H Saltzman WM Chemotherapeutic drugs released from polymers: distribution of 1,3-bis(2-chloroethyl)-1-nitrosourea in the rat brain Pharm Res 1996 13 671 682 10.1023/A:1016083113123 8860421

[B14] Billiau H Heremans D Ververken J van Damme H Carton P de Somer Tissue distribution of human interferons after exogenous administration in rabbits, monkeys, and mice Arch Virol 1981 68 19 25 10.1007/BF01315163 6166278

[B15] Baskin DG Woods SC West DB van Houten M Posner BI Dorsa DM Porte D Immunocytochemical detection of insulin in rat hypothalamus and its possible uptake from cerebrospinal fluid Endocrinology 1983 113 1818 1825 10.1210/endo-113-5-1818 6354698

[B16] Day-Lollini PA Stewart GR Taylor MJ Johnson RM Chellman GJ Hyperplastic changes within the leptomeninges of the rat and monkey in response to chronic intracerebroventricular infusion of nerve growth factor Exp Neurol 1997 145 24 37 10.1006/exnr.1997.6448 9184106

[B17] Yamada K Kinoshita A Kohmura E Sakaguchi T Taguchi J Kataoka K Hayakawa T Basic fibroblast growth factor prevents thalamic degeneration after cortical infarction J Cereb Blood Flow Metab 1991 11 472 478 2016355 10.1038/jcbfm.1991.90

[B18] Nutt JG Burchiel KJ Comella CL Jankovic J Lang AE Laws ER Lozano AM Penn RD Simpson RK Stacy M Wooten GF Randomized, double-blind trial of glial cell line-derived neurotrophic factor (GDNF) in PD Neurology 2003 60 69 73 12525720 10.1212/wnl.60.1.69

[B19] Rennels ML Gregory TF Blaumanis OR Fujimoto K Grady PA Evidence for a 'paravascular' fluid circulation in the mammalian central nervous system, provided by the rapid distribution of tracer protein throughout the brain from the subarachnoid space Brain Res 1985 326 47 63 10.1016/0006-8993(85)91383-6 3971148

[B20] Szentistvanyi I Patlak CS Ellis RA Cserr HF Drainage of interstitial fluid from different regions of rat brain Am J Physiol 1984 246 F835 F844 6742132 10.1152/ajprenal.1984.246.6.F835

[B21] Oldendorf WH Why is cerebrospinal fluid? Bulletin of the Los Angeles Neurological Society 1967 32 169 180

